# What Function Do Platelets Play in Inflammation and Bacterial and Viral Infections?

**DOI:** 10.3389/fimmu.2021.770436

**Published:** 2021-12-14

**Authors:** Beata Tokarz-Deptuła, Joanna Palma, Łukasz Baraniecki, Michał Stosik, Roman Kołacz, Wiesław Deptuła

**Affiliations:** ^1^ Institute of Biology, University of Sczecin, Szczecin, Poland; ^2^ Department of Biochemical Sciences, Pomeranian Medical University, Szczecin, Poland; ^3^ Institute of Biological Science, Faculty of Biological Sciences, University of Zielona Góra, Zielona Góra, Poland; ^4^ Institute of Veterinary Medicine, Faculty of Biological and Veterinary Sciences, Nicolaus Copernicus University in Toruń, Toruń, Poland

**Keywords:** platelets, immunity, inflammation, bacterial infection, viral infection

## Abstract

The article presents the function of platelets in inflammation as well as in bacterial and viral infections, which are the result of their reaction with the endovascular environment, including cells of damaged vascular endothelium and cells of the immune system. This role of platelets is conditioned by biologically active substances present in their granules and in their specific structures – EV (extracellular vesicles).

## Introduction

It is currently assumed that mammalian platelets, in addition to participating in the coagulation processes and maintaining blood vessel integrity, or ensuring homeostasis and intravascular resistance, are effector elements of immunity and inflammation with modulatory function, involved in physiological and pathological conditions (1–50). This role of platelets results from and is related to their reactivity formed as a consequence of the reaction between their receptors located mainly on the surface, and vascular endothelial cells, specifically the subendothelial layer in the damaged blood vessel part. This results in their changed metabolism and activation, manifested by their increased recruitment, adhesion, aggregation and secretion, including the reorganisation of their cytoskeleton, leading to a change in their shape, including an increase in their surface area. Platelets in such a state, inducing secretion of biologically active substances from membrane granules and EV (extracellular vesicles), cause a change in the activity of endothelial cells of blood vessels, as well as a change in the activity of cells of the blood immune system (8–10, 12, 22, 25, 27, 28, 33, 34, 39–43, 48, 51–59). As a result of this condition, activating substances secreted from platelets and leukocytes, including immunity-related substances and antimicrobials, determine not only the immune status in the vessels, but also activate blood coagulation and clot formation (6, 9–11, 17, 25–28, 30, 33–35, 38, 44, 48, 49, 59–65). As a result, acting through plasma "factors", they participate in the first and second wave of vascular homeostasis, the so-called "wave of protein homeostasis". In addition, platelets "controlling" the endothelium of blood vessels in a "touch and go" manner, including circulating blood leukocytes, are basically constantly "exposed" to activation, for example as a result of the effects of vWF (von Willebrand factor), TxA2 (thrombin, thromboxane A2), P-selectin or collagen (15–17, 34, 42, 46, 50, 62). Such a vessel-located platelet function, along with the ability to recognise PRRs (pathogen recognition receptors) and participation in defence processes, e.g. in the NET (Neutrophil Extracellular Traps) network, phagocytosis, as well as antigen presentation together with professional antigen-presenting cells, make them important elements of the intravascular immune barrier, including barrier against infections, from the moment of the " pathogen "concentration" to their direct and (or) indirect elimination (6, 8–12, 16, 21, 23–28, 30, 31, 34, 35, 37, 40, 42–49, 56, 62, 63, 65–67). Having considered the foregoing, platelets, thanks to constant surveillance and "scanning" of the blood vessel environment, including damaged layers of the extracellular matrix of vascular endothelial cells and immune cells in the blood, are elements that supervise vascular regulation (1–15, 17–20, 30–38, 41–46, 48, 50, 53, 59–66, 68–77). As a result, they are the guards of homeostasis, innate and acquired intravascular immunity in physiological and pathological states, including infectious and non-infectious diseases as well as autoimmune diseases and neoplastic processes.

## Platelets and Inflammation

In vertebrates, the immune system has evolved by forming immune cells, which are elements in case of which, platelets – cells with a remarkable ability to change activity, including anti-inflammatory and immune activity, also associated with their morphological changes – also can be included, due to their functions and roles (1–14, 17, 24–26, 34, 44, 58). In physiological conditions, the platelet count increases as a result of the reactivity of progenitor megakaryocytes, and during infection, additionally as a result of the activity of the released pro-platelet cytokines (10–12, 17, 34). When the integrity of blood vessels is disturbed and inflammation occurs in vertebrates, e.g. due to a thromboembolic disease, defined as DIC (disseminated intravascular coagulations), the first cells recruited by the vascular endothelium are platelets (4, 9–11, 17, 25, 26, 34, 35, 43, 45, 46, 49, 78, 79). As a result of the reaction of their markers located mainly on the surface (selectin and integrin receptors, including HPA (Human Platelet Alloantigens), C-type lectin or C-type lectin-like receptors, TLR (Toll like-receptors), FcɣRIIa (immunoglobulin Fc receptor), CR (complement receptor), cytokines and chemokines, CD (Cluster of Differentiation), ADP (Adenosine diphosphate receptor), P2Y (Purinoreceptor) and PAR-1 and 4 (Protease-activated receptors 1 and 4), specifically with the subendothelial layer of the damaged and exposed endothelium of blood vessels vWF, collagen, thrombin, fibronectin, laminin, vitronectin, receptor TLR, NLR (nucleoide-binding oligomerisation domain-like receptor), they are activated and interact with the blood vessel environment, including PMNs (polymophonuclear cells), MNs (mononuclear cells), DC (dendritic cells), T and B cells, takes place, which leads to inflammation (2, 8, 11, 12, 18, 19, 24, 27, 34–36, 38, 41, 42, 44, 46–49, 66, 67, 74, 77, 78, 80–83). As a result of the activation of platelets, they release numerous biologically active substances from their granules (**Table 1**), including Weibel-Palade bodies and EV (extracellular vesicles) (21, 22, 25–28, 34, 39, 51, 52, 60, 66, 73, 90–92). These substances include growth factors and mitogens, cytokines and chemokines, amines and mediators of various enzymes, HSPs (Heat shock proteins), C8 group tetraspanin proteins associated with ADAM molecules (a disintegrin and metalloproteinase), pro-apoptotic proteins, as well as microRNA, mRNA, lipids and other compounds, creating transcription, regulatory, activating and angiogenic factors.

**Table 1:** Selected biological platelet factors (1–4, 6, 18, 20, 24, 42, 53, 54, 56, 57, 60, 78, 84–89).

Granules and platelet elements	Substances found in platelet granulations:
α Granules	**1. growth factors and mitogens**: *TGF-β, *PDGF, *EGF, *IGF, *VEGF, *HGF, *FGF, *GM-CSF, *PD-ECGF, endostatins; **2. coagulation system proteins**: fibrinogen, *vWF, integrins, factors V, VII, XI, XII, XIII, heparin-binding platelet factor h, protein S, kininogens, plasminogen, thrombin, alpha 2-antiplasmin; **3. cytokines:** IL-1, IL-1α, IL-1β, IL-6, IFN-γ TNFα, *MIF, *MMP 1, 2, 3, 9 i 14; **4. chemokines (kinecidines)**: *PF-4 (CXCL4), IL-8 (CXCL8), *RANTES (CCL5), β-thromboglobulin (CXCL1), *SDF-1 (CXCL12), *MIP-1α (CCL3), *TARC (CCL17), *ENA-78 (CXCL5), *MCP-1 (CCL2), *NAP-2 (CXCL7), *GRO-α (CXCL1), *CXCR4, *CCR4; **5. adhesion molecule**: vitronectin, fibronectin, thrombospodin, *β-TG; *ICAM-1, *VCAM-1, selectin P (CD62P) and E and *CD40L (CD154), *TLT-1; **6. inhibitors**: *TIMP, *ADAM, α_2_-macroglobulin, α_2_-antitrypsin, α_2_-antiplasmin, antithrombin, *PAI-1, inhibitor C1, *COX-2, membrane glycoproteins, *TFPI, protein S and C, nexins (amyloid protein betaA4), plasmin proteases, *HMWK; **7. other molecules**: defensins, PmP*, β-lysine, IgG, IgE, IgM, albumin, transferrin, angiopoetin 1, 2, 3, *APP, thymosin β-4, fibrinopeptide A i B, *TRAP, *HMBG-1, *TXA2, *PAF;
Osmophilic granulations (δ – dense granules)	**1. amines and mediators:** thrombin, serotonin, histamine, catecholamines (noradrenaline/adrenaline), epinephrine; **2. nucleotides**: *ATP, *ADP, *GDP; **3. other molecules:** Ca^2+^ and Mg^2+^ ions, selectin P, glutamates, thrombocidins 1 and 2, poly-phosphatases, pyrophosphates;
Lysosomes	**1. proteolytic enzymes:** carboxypeptidases A and B, cathepsin A, D and E, colagenase, acid phosphatase, aryl sulphatase; **2. glycohydrolases:** heparinase, β-glucoronidase, arylsulfatase, β-galactosidase, P-glycerophosphatase, β-glucosidase, D-glucosidase, β-fucosidase, α-mannosidase, D-mannosidase
Peroxisomes	**catalase, peroxidase**

The symbol * is assigned to substances whose full names are explained below the table. ADAM, a disintegrin and metalloproteinase; ADP, adenosine diphosphate; APP, amyloid precursor protein; ATP, adenosine triphosphate; β-TG, beta-thromboglobulin; COX, cyclooxygenase; CCR4, C-C chemokine receptor type 4; CD40L (CD154), cluster of differentiation; CXCR4, C-X-C chemokine receptor type 4; EGF, epidermal growth factor; ENA-78, epithelial neutrophil-activating protein 78; FGF, fibroblast growth factor; GDP, guanosine diphosphate; GM-CSF, granulocyte-macrophage colony-stimulating factor; GRO-α, growth-regulated α; HGF, hepatocyte growth factor; HMBG-1, high mobility group box 1; HMWK, high-molecular-weight kininogen; ICAM-1, intracellular adhesion molecule; IGF, insulin-like growth factor; MCP-1, monocyte chemotactic protein 1; MIP-1α, macrophage inflammatory protein 1α; MIF, macrophage migration inhibitory factor; MMP, matrix metalloproteinases; NAP-2, neutrophil-activating peptide-2; PAF, platelet-activating factor; PAI-1, plasminogen activator inhibitor 1; PD-ECGF, platelet-derived endothelial cell growth factor; PDGF, platelet-derived growth factor; PF-4, platelet factor 4; PmP, peroxisomal membrane protein; RANTES, regulated on activation, normal T expressed and secreted; SDF-1, stromal cell-derived factor-1; TARC, thymus and activation-regulated chemokine; TFPI, tissue factor pathway inhibitor; TGF, transforming growth factor; TIMP, tissue inhibitors of metalloproteinase; TLT-1 (TREM - triggering receptor expressed on myeloid cells) - like transcript-1; TRAP, tryptophan regulated attenuation protein; TXA2, thromboxane A2; VCAM-1, vascular cell adhesion molecule 1; VEGF, vascular endothelial growth factor; vWF, von Willebrand factor.

These substances of platelets, activating vascular endothelial cells, cause a change in the permeability and tension of blood vessels, which determines the integrity in the places of damage to these vessels, and by the interaction with the immune cells of the blood, they change their reactivity. This leads to intravascular activation (11, 12, 17, 34, 43, 48, 49, 62, 83, 91). These platelet-derived substances, including chemokines CCL3 (MIP1 α-macrophage inflammatory proteins 1α), CCL5 (RANTES) and CXCL4 (PF-4 -platelets factor 4), interacting with blood leukocytes as well as platelets during the stages of the extravasation cascade, coordinate their recruitment and activation (17, 19, 25, 62, 85). In this condition, the exposed collagen in the damaged endothelium of blood vessels, resulting from injury or colonisation of bacteria and viruses, after binding to the GPVI receptors of platelets, induces the recruitment of leukocytes and platelets and their activation, which causes inflammation (10, 17, 18, 30, 33, 34, 42, 48, 62, 93). In addition, the activation of platelets is enhanced by P-selectin secreted by cells of the damaged endothelium of blood vessels, which also stimulates platelets and activates mainly neutrophils and monocytes towards the activation of endothelial cells of blood vessels (2, 8, 10–12, 34, 41, 42, 48, 61, 69, 78, 94). It should be added that such a proinflammatory effect of activated platelets is also associated with the secretion of PAF (platelet-activating factor) and VEGF (vascular endothelial growth factor), which cause the relaxation of the endothelium of blood vessels and an increased inflow of leukocytes and platelets, which leads to the development of inflammation (6, 8, 21, 33, 35, 48, 66, 78, 86). These activated platelets are characterised by increased expression of ICAM-1 (intracellular adhesion molecules) and MCP-1 (monocyte chemoattractant protein-1), which cause increased adhesion of neutrophils and monocytes as well as increased inflow of dendritic cells and increased inflammation (4, 8, 9, 41, 65, 77, 78). Platelets in such a state, affecting, among others, neutrophils, increase the production of leukotrienes (LTC4, LTD4, LTE4), which subsequently increases vascular permeability and is the cause of exudate and oedema formation (6, 35, 48, 95). Furthermore, the effect of activated platelets on neutrophils in the NET (Neutrophil extracellular traps) network with the participation of platelets and the process of PMN cell apoptosis determining the lifetime of these cells, additionally influences inflammation (11, 12, 22, 23, 35, 58, 69, 95). The activation state of platelets also includes the change of their shape from discoid to circular, with numerous pseudopodia (10, 16–19, 40, 42–44, 96). In the case of activation of platelets as a result of a bacterial infection, these cells, among others, affect the CXCL4 (PF-4) and CCL5 (RANTES) chemokine receptors on the surface of the vascular endothelium, induce "extravasation" of leukocytes (29, 30, 38, 62, 81). Their cooperation with monocytes induces the formation of a functional receptor – the NLRP3 inflammasome (nod-like receptor protein 3), which results in the secretion of the proinflammatory cytokine IL-1ββ (interleukin 1β) and IL-18 (10, 12, 17, 32, 37, 42, 78, 97). On the other hand, in the case of viral infection, activated platelets, interacting with monocytes, cause immuno-metabolic reprogramming of these cells, e.g. in the area of the biogenesis of lipid droplets– functional and active elements in the body in its physiological and pathological state (40). Furthermore, activated platelets, together with calcium and magnesium ions, can cause clots and even the formation of thrombi in the blood vessels, which can also cause inflammation (3, 6, 11, 12, 17, 61, 85, 98). Regardless of the reason for the activation of platelets, such a state each time arises as a result of specific and activating signalling pathways present in these cells, in terms of, among others, the immunoreceptor tyrosine-based activation motif, including tyrosine kinase – Scr (phosphoinositide S-kinase), protein kinase C and A and lipid kinase, as well as activation of G protein (4, 6, 8, 10, 11, 35, 42, 45, 49, 50, 61, 62, 78, 83, 99, 100). As a result, the homeostatic system in blood vessel changes from thromboembolic condition (clot) to a pathological condition such as thrombosis.

Platelets involvement has been demonstrated in numerous conditions, due to their broad role in the microorganism, e.g. in modulating processes in blood vessels, tissue regeneration, as well as interaction with PMN, MN, B, T, NK (natural killer cells), DC cells and epithelial cells, as well as their rapid transport to sites of injury or infection to repair damage and (or) recognise and neutralise pathogens (43–45, 47, 48, 50). Their involvement has been reported in bacterial and viral infections (see further chapters of this paper), sepsis, stroke, neoplastic process - cancer, rheumatoid arthritis and autoimmune diseases, diabetes and obesity, spleen and kidney injuries, diseases of the CNS (central nervous system), myocardial infarction and myocarditis, asthma, pneumonia, atherosclerosis and vascular diseases, as well as inflammation of the mouth, large intestine and kidneys (reference in the text). Such diverse roles of platelets have resulted in the fact that recently subpopulations based on their function have been distinguished (50).

In the case of sepsis, the role and participation of platelets in this condition was determined based on the decreased activity resulting from the deficiency of their GPIbβ receptor - a fibrinogen-specific marker, which is manifested by a weaker interaction of platelets/neutrophils and platelets/monocytes, including reduced synthesis of proinflammatory factors such as: TNF-α (Tumour necrosis factor-α), MCP-1, IL-6 and MIP-1β, which in turn reduces the immune status of the macroorganism and increases susceptibility to infection (34, 76, 78). This medical condition is also associated, as a result of reduced expression of platelet receptors for C1q, C3a and C5a components of the complement system, with a decrease in their activity and changes in the recruitment of cells of the immune system (34). The result is the inhibition of innate immunity related to e.g. the complement system, although this condition also causes an increased release of TF (tissue factor) and vWF, which enhance the inhibition of coagulation and coagulation cascade - elements important in inflammation (34, 46, 101).

In turn, the stroke-related role of platelets in humans results from the fact that they connect to the endothelium of blood vessels, thus creating a bridge for leukocytes flowing through and cause inflammation, although it has been shown that even their aggregation, which inhibits perfusion, is an additional potential tissue damaging element (2, 35, 76, 98, 102). During this medical condition, platelets stick to the surface of the endothelium of the brain's blood vessels, where there are few adhesive factors, and are able to produce a much larger amount of ligands, including those for P-selectin or integrins, which increases the recruitment of leukocytes and contributes to the formation of inflammation (6, 30, 42, 76). It has been described that activated platelets during stroke show high expression of CD40 receptors, which results in increased recruitment of T cells, which interact with platelets to induce secretion of biologically active substances from their granules, e.g. vWF, which is an important factor contributing to immune thrombocytopenia mediated by platelet GPIb integrin receptors (46).

In the case of the neoplastic process or cancer, it has been shown that platelets, as a result of the interaction, mediated by TLR-4 receptors, with neoplastic cells, secrete substances from α granules, such as HMGB1 (high mobility group box 1) protein and TNF-β, which leads to the activation of the immune system and enhancement of inflammation, but also to an increase in the invasiveness of cancer cells (76). The condition of increased potential for cancer metastasis has also been found when increased platelet aggregation has been registered, as this condition, caused by the activity of, among others, a member of the metalloproteinases family (MMP - matrix metalloproteinases) - ADAM-10 (a disintegrin and metalloproteinase) and the NLRP3 inflammasome, increases inflammation (12, 14, 76, 103, 104). It has also been reported that cancer cells, by secreting thrombin, transform fibrinogen into fibrin (12, 60, 105). Also activation of platelets through PAR receptors and coagulation factors V, VIII, IX and XIII, stimulate them to secrete a CXCL7 chemokine (NAP-2 - neutrophil-activating peptide-2) and a member of the MMP protein family-ADAM-10, leading to increased recruitment of neutrophils and T cells and their higher activity as well as activation of inflammation (9, 32, 60, 103, 104). It has been reported (12, 60, 76) that during the neoplastic process related to cancer, platelets "cover" cancer cells, and thus protect them against the activity of NK cells, thereby lowering the immune status and reducing inflammation. Such a situation, or reduced macroorganism defensive reactivity, may also arise as a result of the secretion of the TGF-β (Transforming growth factor β) cytokine by platelets, which, as a result of reduced mobility of NK cell granules, decreases the cytotoxicity of these cells (62). Due to impaired secretion of IFN-γ (interferon gamma) and the C8 tetraspanins, reduces the activity of the components of the MMP protein family – ADAM-10 (12, 37, 60, 76). Moreover, TGF-β cytokine, by inhibiting the activity of cytotoxic T lymphocytes and affecting the function and differentiation of Treg cells, also reduces the immune status, including anti-cancer and anti-inflammatory status (62). It has been demonstrated that the neoplastic process may also mask the MHC class I (Major histocompatibility complex I) receptors of platelets carried by cancer cells, which also reduces their immune response and thus reduces inflammation (12, 76).

Platelet involvement has also been reported in rheumatoid arthritis, although they way how they penetrate through the synovial endothelium into the joint and increase inflammation is not entirely clear (76). It is assumed (48), that they are either transferred using surface receptors to the synovial part of the joints by adhesion to migrating leukocytes, or they penetrate there as a result of "leaking" synovial endothelium (76). Considering the first pathway, it must be stated that although there are many leukocytes with platelet markers in the inflamed joint, their detection does not constitute any evidence, because it is known that platelet-leukocyte interactions are initiated as early as in the blood. On the other hand, the evidence of vessel "leakage" may be related to the existence of small holes between the endothelial cells, which allows fluid and particles to escape from the vessels, letting them travel to the synovial space. Doubts about the latter hypothesis are related to the fact that the openings in the vascular endothelium are too small to "pass" platelets through, hence it is assumed that there is no certain evidence that they use this pathway (2). It is assumed (106) that platelets themselves do not have direct access to the synovial membrane of joints, but it is accessible by their surface "platelet-derived microparticles", currently referred to as EV. These elements account for much as 70-90% of those elements in the blood, that are rich in biologically active substances such as cytokines, chemokines, HSPs, nucleic acids (microRNA and mRNA), as well as lipids, which not only activate the immune system and angiogenesis (21, 22, 25–28, 34, 39, 51, 52, 60, 66, 73, 90–92), but also the synovial membrane of the joints (51–55, 60, 91). Thus, if the EVs of platelets, which are much smaller than these cells, enter through microopenings in the vascular endothelium, they also precede the appearance of leukocytes in the synovium, which is another fact to refute the hypothesis that leukocytes "lift" platelets from the lumen of the blood vessels. Hence, it is assumed that the EVS of platelets reaching the synovial membrane of joints and accumulating inside, connect the leukocytes recruited there into aggregates using the integrin receptor of GPV and FcɣRIIa of platelets, and additionally, activated with P-selectin, increase the recruitment of leukocytes to the vascular membrane of the joints, which together with the effect of IL-1, IL-6 and TNF-α on blood vessels and synoviocytes, increases the formation of arthritis (2, 10, 18, 52, 56, 76, 85, 91).

The involvement of platelets has also been found in autoimmune diseases, because in the case of multiple sclerosis in humans, it has been shown that platelets, by inducing inflammation, lead to thrombocytopenia and reduce the symptoms of this disease (16, 68, 102). The condition of thrombocytopenia resulting from inhibition of an inflammatory marker - CD40 marker on platelets (84, 107), was also recorded in mice suffering from systemic lupus, which resulted in reduced symptoms of this disease and increased survival of the animals (2, 51, 52). Platelets reduce the inflammatory process also in other autoimmune diseases (2, 35, 107), in which not only thrombocytopenia, but also reduced recruitment of blood leukocytes, which limited the formation of oedema - one of the basic symptoms of inflammation, have been found (78).

It has been shown that in diabetes and obesity as well as in spleen or kidney failure and CNS diseases, as a result of low reactivity of platelet receptors, their dysfunction occurs, which leads, among others, to blood clotting disorders (8, 20, 76, 80), causing blood vessel embolism (16, 22, 76, 92, 108) and thrombosis (10, 11, 20, 30, 42, 62, 64, 76, 80, 99, 109). The role of platelets has also been demonstrated in myocardial infarction, because the change in their activation mediated by PAR-1 and P2Y12 receptors in terms of, e.g., aggregation, leads to increased blood clotting and the formation of a thrombus composed mainly of fibrin, platelets and a large amount of vWF (19, 46, 85, 110–112). This causes coronary occlusion and may lead to atheromatous plaque rupture and inflammation. The involvement of platelets was also recorded in myocarditis caused by the infections with entero-, adeno- and herpesviruses, parvovirus B19, HIV and influenza virus, as they enhance the immune status and inflammation if activated by these infectious agents, acting together with the cells of the blood immune system (83, 113). Moreover, HIV infection has been shown to increase the amount of EV of platelets and their products, including the activity of IFNɣ and IL-1α, which also increases inflammation (113). The involvement of platelets was also found in patients with asthma, pneumonia, atherosclerosis and vascular diseases, as well as inflammation of the mouth, large intestine and kidneys, which are conditions caused, among others, by the interaction of these cells with monocytes, which leads to an increase in the platelet/monocyte complexes and increased secretion of cytokines activating the immune state and inflammation (6, 15, 33–35, 40, 66, 76, 78, 98, 102, 114, 115).

## Platelets and Bacterial Infections

**Figure 1:**
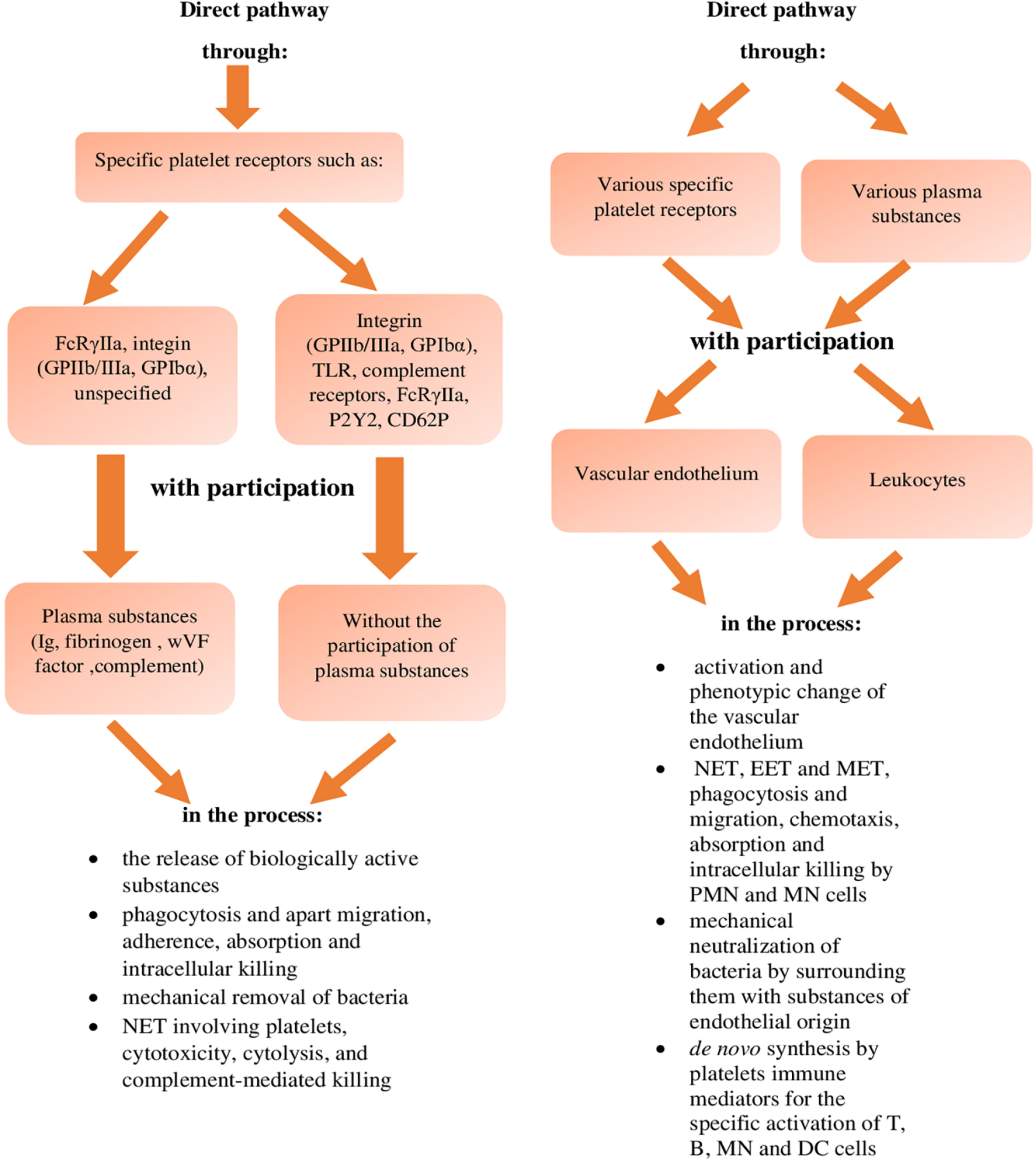
Effect of inactivating platelets against bacteria (reference in the text). NET, Neutrophil Extracellular Traps; EET, Eosinophil Extracellular Traps; MET, Monocyte-drive Extracellular Traps; PMN, polymorphonuclear cells; MN, mononuclear cells; DC, dendritic cells.

The antimicrobial role of platelets was mentioned as early as over a hundred years ago, and in the early 1960s, their activity against bacterial toxins was demonstrated (1, 2, 20, 87). In the following years, studies in this field concerned the impact of yeast on the process of platelet aggregation (1, 2, 8), while the exact role of platelets in bacterial infections for *Gram-positive* bacteria, and then for *Gram-negative* bacteria was described a little later (1–3, 8, 20, 21, 87, 95, 116). It is currently assumed (43) that the involvement of platelets in bacterial infections, in order to eliminate or reduce these pathogens, is associated with their direct effect (**Figure 1**), or interaction between specific platelet receptors and bacteria with no plasma substances and with the involvement of specific plasma substances. In turn, the indirect pathway of the destructive effect of platelets on bacteria (**Figure 1**) occurs as a result of activation of vascular endothelium and/or blood leukocytes by platelets, mediated by various receptors and (or) various plasma substances, in order to activate them towards the neutralising effect on bacteria (38, 43, 59).

In the case of direct neutralisation of bacteria by platelets, with no involvement of plasma substances (**Figure 1**), these reactions take place between the platelets and bacteria, using their specific receptors, or: - integrin markers GPIbα and GPIIb/IIIa (αIIbβ3) - TLRs, - complement markers (38, 43, 44, 59), - and possibly FcRɣIIa, P2Y and CD62P receptors (43, 45, 59). During the direct connection binding of bacteria by platelets mediated by the GPIbα and GPIIb/IIIa (αIIbβ3) integrin surface receptors, this reaction was observed during the infection by *Streptococcus (S) pyogenes*,

S. pneumoniae, S. mutant, S. agalactie, S. sanguinis, S. gordonii and Staphylococcus (Staph.) aureus and Staph. epidermidis as well as Borrelia (B.) burgdorferi, B. hermii (1, 8, 10, 11, 20, 31, 33, 38, 43, 59, 88, 95, 111, 117). Some S. sanguinis and Staph. aureus strains bind to platelets only through the gC1q-R/P33 complement receptor (1, 8, 10, 11, 29, 59, 87). The latter way of platelet association mediated by this receptor, during Staph. aureus infection, additionally leads to the presentation of these bacteria to DC cells for transfer to cytotoxic T lymphocytes (10).

In turn, the pathway of direct association of platelets with bacteria mediated by TLRs was found during infection with various streptococci (43), including *S. pneumoniae* (59). In this pathway of direct neutralisation of bacteria by platelets, mediated by TLRs, in the case of *Gram-positive* bacteria, their lipoteichoic acid is bound by the platelet TLR2 receptor, while in the case of *Gram-negative* bacteria, their LPS (lipopolysaccharide) is bound by the platelet TLR4 marker (8, 20, 31, 44, 59, 95, 111). The direct binding of bacteria by platelets mediated by TLRs is different in humans and mice, because in humans this interaction occurs through the TLR4 receptor, and in mice through the TLR5 and TLR9 markers, and additionally, the bactericidal substances in mice are defensins and microbicidal proteins PMPs (peroxisomal membrane proteins), while in humans, they additionally include β-lysine and β4 thymosin (8, 10, 11, 20, 69, 71, 87).

However, during this direct pathway of neutralising bacteria by platelets, but with the involvement of plasma substances (**Figure 1**), this interaction is performed using additionally their specific receptors, which are: - FcRɣIIa marker, - GPIIb/IIIa and GPIbα integrin marker, - unspecified receptors which bind in a specific sequence to specific plasma substances, that is: - immunoglobulins, - fibrinogen and vWF, and complement components (38, 43, 59), which was found, among others, during the infection with *Staph. epidermidis* and *Staph. aureus* and *S. pneumoniae*, *S. pyogenes, S. agalactie, S. mutans, S. sanguinis, S. gordoni and S. oralis* as well as *Helicobacter pylorii* and *Porphyromonas gingivalis* (29, 59, 87).

It is assumed that in the direct way of neutralising bacteria by platelets, or with the involvement of their specific surface receptors, with no involvement of plasma substances and with their involvement, this pathway of the destructive effect of platelets on bacteria occurs through (**Figure 1**):

- secretion of biologically active substances
- the process of phagocytosis and separately the process of migration, adherence, absorption and intracellular killing
- mechanical removal of bacteria
- the NET network with the involvement of platelets, cytotoxicity, cytolysis and complement-related bactericidal properties (references in the text).

The first pathway of direct neutralisation of bacteria by platelets thanks to the secretion of bactericidal substances contained in their α-granules, is associated with defensins, microbicidal PMPs, β-lysine, thymosin β-4 and β-5, platelet basic proteins - NAP-2 (CXCL-7), kinocidins, or chemokines with bactericidal activity CXCL4 (PT-4), CCL3 (MIP-2β) and CCL5 (RANTES) and IL-8 (CXCL-8), fibrinopeptides A and B, reactive oxygen species (ROS) and connective CTAP-3 (tissue activating peptide 3) (8, 10, 11, 17, 20, 25, 34, 35, 37, 38, 42, 44, 62, 69, 71, 87, 88, 118) as well as cathelicidins and granulysins (119). It has been shown that during infection with α-toxin of

*Staph. aureus*, this direct pathway of neutralising bacteria by platelets is mainly caused by the action of β-defensins and PMPs, the substances which not only sequester these pathogens, but also activate them to form the NET network involving platelets (42, 44, 71). The role of these bactericidal substances has also been demonstrated in infections with *Clostridium* sp., *Microccocus* sp., *Lactobacillus* sp. as well as *Listerii monocytogenes* and *E.coli* (2, 8, 10, 11, 25, 71), however, the secretion of β-defensins by platelets during these infections occurs through the formation of pores, and not through the tubules of platelets (10, 11, 71). Furthermore, bactericidal substances in this direct pathway of platelet effect on bacteria are class G and M immunoglobulins present in their α-granules (**Table 1**). In the case of infections with *Bacillus subtilis, Lactococcus lactis* and

*Staph. aureus* (3, 4, 20, 21), the direct neutralisation of bacteria by platelets results from the action of thrombocidins 1 and 2 present in their osmophilic granules (also known as truncated variants of CXCL chemokines, or the NAP-2 peptide - CXCL-7) (44), and cathepsin A, D and E (20, 44, 120), as well as proteolytic enzymes found in their lysosomes (21), in addition to catalase and peroxidase present in their peroxisomes (**Table 1**).

On the other hand, the second direct pathway of the platelet effect on bacteria through the process of phagocytosis of these cells occurs with the involvement of their surface integrin receptors - GPIIb/IIIa (11, 34, 45, 48, 63), although in the case of periodontal bacteria this process occurs with the involvement of TLR-2 receptors (63), and in the case of unspecified bacteria with the involvement of FcRɣIIa receptors (34). It has been shown (43) that the process of platelet phagocytosis mediated by TLR-2 receptors additionally induces them to contact PMN cells with the involvement of the CD62P selectin receptor (P-selectin) and integrin GPIIb-IIIa. It has been recorded that the bacteria phagocytosed by platelets are only sequestered in the phagocytic vacuoles in this process, but are not killed as a result of the lack of strong oxidation and lowered pH (100), although in the case of *Staph. aureus*, PMPs and kinocidins present in the phagocytic vacuole degrade these bacteria (43). This pathway of direct neutralisation of bacteria by platelets also includes the interaction with platelets based on their ability to migrate, adhere, absorb, and also intracellularly kill as a result of the production of ROS reactive oxygen species (8, 10, 11, 17, 21, 23–25, 34, 37, 41, 42, 48, 58, 59, 78, 100, 110, 121–124). This direct destructive effect of platelets on bacteria, separately caused by migration, adherence and absorption of platelets, was recorded during infection with *Bacillus* sp.*, Clostridium* sp.*, Micrococcus* sp.*, Lactobacillus* sp.*, Staph.* sp.*, E. coli*, *Helicobacter pylorii* as well as unspecified bacteria (17, 21, 25, 48, 64, 121). It has been demonstrated (23, 24, 41, 124) that absorption capacity of *Staph. aureus* by platelets is enhanced by the aggregation of these cells, and it has been shown that in the case of this bacterium, as well as *E. coli*, this reaction is slightly faster than in the case of PMN cells absorption capacity (21).

In turn, the third pathway of direct platelet effect on bacteria, based on their mechanical removal, is related to the ability of platelets to move, as a result of their autocrine activation, which increases their migration and leads to "scavenging" lost bacteria from blood vessels (45, 82, 121). It has been described that during such mechanical removal of *Gram-negative* bacteria by platelets, their binding to these pathogens is mediated by the TLR-4 receptor, analogously to the formation of a clot, although the course of the latter reaction is different (8, 10, 11, 15, 31, 111). On the other hand, the association of platelets with *Borrelia* sp., in this direct way of neutralisation, is mediated by the αIIbβ3 integrin receptor (43). The immobilisation of *Listeria monocytogenes* by the formation of aggregates of platelets and these bacteria, which is mediated by the complement receptor C-3 and the GPIba integrin marker (62, 77), also belongs to the direct pathway of platelet activity, or the mechanical removal of bacteria (62, 77). Furthermore, this process of mechanical removal of bacteria by platelets involves the immobilisation and removal of bacteria by the formation of platelet/bacteria aggregates (17, 21, 24, 87, 124), resulting from the "opsonisation" of bacteria with platelet fibrinogen and platelet TLRs and GPIIb/IIIa receptors (34). This pathway of neutralising bacteria by platelets also includes the removal of these pathogens by their hermetisation performed by enclosing, encapsulating and binding platelets with pseudopodia (phyllo- and lamellipodia), which has been observed in infections with *Staph. aureus* and *E.coli* (62, 100). The latter bacteria neutralisation method leads to the formation of "clusters" of these pathogens that can be presented to professional phagocytic cells (62, 100). It has also been described, but based on *in vitro* studies (125), that this way of mechanical removal of *Leptosira interrogans* by platelets is also the aggregation of these bacteria with platelets, resembling hemophagocytosis (100), which, as reported (125), is ineffective because platelets are decomposed by the toxins of these bacteria.

In the case of the fourth way of the direct effect of platelets on bacteria, e.g. *E. coli* and *Staph. aureus* in the NET network involving platelets, it has been shown that in this process, platelets bind to these pathogens *via* the TLR-2, FcRɣIIa, P2Y6 (43) and CD62P (126) receptors. It has been reported that in this process platelets intensely destroy bacteria, and it has been found that this reaction is more than 10 times faster than the action of the NET network with no involvement of platelets (10, 11, 17, 33, 34, 45, 58, 62, 95, 100, 127). It has been described that during the neutralisation of bacteria by platelets in the NET network involving platelets, TxA2 (thromboxane A2) is also released, which *via* the TxA2 receptors of vascular endothelial cells (43), additionally activates platelet integrin receptors towards their aggregation as well as affects vascular permeability and PMN cell recruitment, which in turn leads to an increase in the effectiveness of the NET network (34). It was has been reported that PMN cells involved in the NET network and platelets interact with each other (62, 126), as demonstrated during sepsis of bacterial origin (42, 116). It has been found that substances secreted by platelets not only activate the killing power of PMN cells against bacteria "captured" in the NET network (2, 126, 127), but also stimulate other blood immune cells towards the secretion of proinflammatory cytokines, thus increasing antibacterial immunity (10–13, 17, 60, 69, 70, 121, 126). This direct pathway of neutralising intracellular bacteria by platelets is part of the cytotoxicity process of these cells, which is related to the presence of IgE receptors on their surface and is conditioned by the activity of the PCIF (platelet cytotoxicity inducing factors) lymphokine secreted by CD4 T cells and PASL platelet activity suppression lymphokines secreted by CD8 T cells (21). The ability of platelets to conduct cytolysis, which is conditioned by serum IgG2a, IgG2b and IgG3 and complement components C5-C9, has been described within this pathway of direct interaction of platelets with bacteria (21, 34). This pathway of neutralising bacteria by platelets also includes their bactericidal properties conditioned by complement proteins, as it has been shown that during bacterial infection an increased expression of the C3a and C5a complement receptors and the release of C8 and C9 complement components from platelets takes place and they create the C5b-C9 protein complex, or the MAC (Membrane Attack Complex), which destroys bacteria by creating transmembrane channels (34, 77). This complex (MAC) also activates vascular endothelial cells to release TF (tissue factor) and vWF, both of which initiate the coagulation cascade, thus additionally creating a physical barrier that immobilises and neutralises bacteria (34).

On the other hand, the indirect pathway of neutralising bacteria by platelets is related to the effect of these cells, through the interaction of their various receptors and various plasma substances with the endothelium of blood vessels and with leukocytes in the blood (38, 43, 59), in order to activate their destructive activity against bacteria, which occurs through (**Figure 1**):

- activation and phenotype change of the vascular endothelium
- activation of the NET, EET (Eosinophil Extracellular Traps) and MET (Monocyte-drive Extracellular Traps) networks, the process of phagocytosis, migration and chemotaxis of PMN and MN cells, as well as the process of absorption and intracellular killing by these cells
- mechanical neutralisation of bacteria by surrounding them with substances of endothelial origin
- *de novo* synthesis of immune mediators by platelets, for the specific activation of T, B cells and MN and DC cells (references in the text).

In the case of the first pathway of indirect effect of platelets on bacteria based on the activation of the vascular endothelium and phenotype change of the vascular endothelial cells, including the subendothelial layer of the tunica intima, it has been shown that it occurs through selectin markers and ICAM - 1 and VCAM - 1 (vascular cell adhesion protein-1) receptors of platelets, leading to increased recruitment of leukocytes, including adhesion of neutrophils to the vascular "network" (1, 2, 8, 10, 11, 15, 33, 58, 62, 127). This condition increases secretion of chemokines by platelets, which results in an increase in the immune status, including antimicrobial immunity. This effect of platelets, leading to the activation of the vascular endothelium, is also associated with the recruitment of neutrophils to areas of vessels with changed phenotype or specific vascular beds, completely devoid of cellular adhesion molecules for PMN cells (1, 11, 33). These actions of platelets take place after binding their GPIb-IX-V receptors to the immobilised vWF in the shielded vascular subendothelium, which activates the adhesion of the platelet glycoprotein – GPVI to collagen (1, 11, 15, 16, 89, 93). Such a condition in damaged blood vessels, including those with phenotype changes, also creates conditions for the formation of an attachment surface for neutrophils, which, *via* PSGL-1 (P-selectin glycoprotein ligand-1), increase inflammation during bacterial infection (1, 11, 127). This pathway also involves the attachment of the C-3 complement receptor and Mac-1 (macrophage 1 antigen receptor) of the endothelium of blood vessels to fibrinogen immobilised on the platelet integrin receptors GPIIbIIIa and GPIbα, which leads to the recruitment of MN cells - important elements in anti-infective immunity, including antibacterial immunity (1, 11). Moreover, the possibility of adhesion of platelets to the vascular endothelium, completely or partially devoid of classical adhesion molecules, also causes the formation of dense adhesive "cushions" for recruitment and "landing" of many leukocytes (62), which is observed in microvascular infections of the brain caused by bacterial infections (1, 10, 11). In addition, activation of the vascular endothelium by platelets, to a phenotype change in the expression of their CD40L receptor, produces an increased amount of adhesion molecules (ICAM1, VCAM1, E-selectin) and chemokines (IL-8, MCP-1-CCL-2) on the vascular endothelium (23, 33, 43, 44). Such a condition enhances the recruitment and adhesion of leukocytes, thereby increasing antibacterial immunity, which is additionally enhanced by substances from platelet EVs and IL-6 and IL-12 secreted by DC cells as well as by products of B and T CD8 cells (23, 33, 43, 44). The phenotype change of the endothelial layer of blood vessels - glycocalyx, leading to a change in its integrity, which causes not only a change in the adhesion and secretion of platelets as well as recruitment and secretion of leukocytes, but also in exocytosis of lysosomes of endothelial cells of blood vessels, containing proteolytic, hydrolytic and glycolytic enzymes that affect endovascular homeostasis, including antibacterial immunity takes also place (17).

On the other hand, the second indirect pathway of neutralising bacteria by platelets, by activating the NET network, occurs with the use of TLR-4 receptors, selectin markers and integrin platelets, and it is very effective, as it additionally affects the secretion of substances enhancing the formation of the NET network by PMN cells (10, 11, 33, 35, 36, 58, 62, 69, 78, 128, 129). This situation, increasing the possibility of the NET network formation during bacterial infection, additionally causes larger recruitment of leukocytes, including PMN cells (8, 58, 62, 71, 95, 126). The confirmation of the role and involvement of platelets in their indirect effect on bacteria, *via* the NET network, is the increased spread of bacteria in mice as a result of the removal of LFA1 (lymphocyte function-associated antigen 1) adhesion particles from platelets, which leads to a weakened interaction of these cells with neutrophils and reduced possibility of the NET network formation (11). These observations in mice also apply to humans, as it has been demonstrated that the formation of the NET network in humans occurs after neutrophils contacting with platelets previously stimulated with the plasma of patients suffering from sepsis caused by *Staph. aureus* and *E. coli* (11, 69). On the other hand, in the case of indirect neutralisation of bacteria by platelets in the EET and MET networks, it has been found that these processes are similar to the neutralisation of these pathogens in the NET network, but in the case of the EET network, it is indicated that its destructive effect is not associated with bactericidal substances present in eosinophil granules, as in the case of neutrophils in the NET network, while in the case of the MET, it has not been shown which cell population - monocytes or macrophages - are more involved in this reaction (45). The process of PMN and MN cell phagocytosis mediated by integrin receptors - GPVI, which was recorded during infection with *Klebsiella pneumoniae*, is also part of this indirect pathway of neutralising bacteria by platelets (71, 130). This indirect pathway of neutralising bacteria by platelets, additionally includes the migration process of PMN and MN cells, activated by IL-8 (CXCL-8) and MCP-1 (CCL-2) secreted by activated platelets during infection with α-toxin from *Staph. aureus* (71, 130). It has also been demonstrated that during unspecified bacterial infections, the HMBG-1 protein derived from α-granules of activated platelets stimulates targeted migration - chemotaxis of PMN and MN cells (76). This pathway of neutralising bacteria by platelets includes the ability to absorb and intracellularly neutralise in an oxygen-dependent bactericidal process performed by PMN and MN cells, as demonstrated in mice suffering from bacterial sepsis (76). It has also been shown that these processes (76) are less effective in relation to the NET network, because the neutralisation of bacteria in the process of absorption and neutralisation performed by PMN cells and Kupffer (MN) cells is four times weaker than in the case of the NET network (8, 11).

The third indirect pathway of neutralising bacteria by platelets is the mechanical neutralisation of these pathogens by surrounding them with fibrinogen from the vascular endothelium, created as a result of the activity of platelets during the surveillance of damaged blood vessels (71, 87). This condition may also create conditions for the survival of bacteria, as has been reported in the case of endocarditis caused by *Staph. aureus*, as the formed aggregates of platelets, bacteria and fibrin, not only protect the microorganisms gathered inside, which can spread (81, 87), but by shielding them against factors used in therapy, damage endothelial cells (20). Such a state can also be recorded when plasma proteins with the NET network create an analogous scaffold for the clot and bacteria (17).

The last, fifth, indirect pathway of platelet interaction with bacteria is their ability to synthesise *de novo* inflammatory mediators, e.g. the chemotactic factor PF4 (CXCL-4) or a functional receptor which is the NLRP-3 inflammasome, determining the specific activation of selected cells of the immune system for the synthesis of IL-1β and IL-18 (2, 11, 17, 51, 97). This condition leads to increased antimicrobial activity of blood leukocytes (23, 33), including CD8 T cells, which was recorded during *Listeria monocytogenes* infection (23, 62). During such a pathway of platelet interaction with bacteria, or the production of substances *de novo*, cTGF-β and IL-1 cytokines are synthesised by platelets *de novo*, which leads to the activation of T cells, including regulatory T cells and B cells in the area of IgA synthesis (62) and stimulating effect on the polarisation of Th1 and Th2 cells (33, 62, 78), towards specific activation of B cells to produce serum immunoglobulins (17). This pathway, or *de novo* synthesis of substances by platelets, includes the production of proinflammatory IL-6, TNFα and chemokines CXCL-8 (IL-8), CXCL-4 (PF-4), CCL3 (MIP-1α) and CCL5 (RANTES), enhancing the recruitment and activation of leukocytes, which was recorded during *Salmonella enterica* infection (11). An analogous picture of *de novo* synthesis of chemokines CXCL-8 (IL-8) and CCL-2 (MCP-1) by platelets was recorded during *Staph. aureus* infection in mice, a condition that promotes increased synthesis of antibacterial substances by monocytes, macrophages and DC cells (71, 130). This route also includes *de novo* synthesis of a substance enhancing the presentation of bacteria to DC cells by platelets (33, 46, 62, 78), as well as substances modulating the cytokine production profile by platelets, affecting macrophages and stimulating them to produce antibacterial substances, which was observed during bacterial sepsis in animals (131, 132).

**Figure 2:**
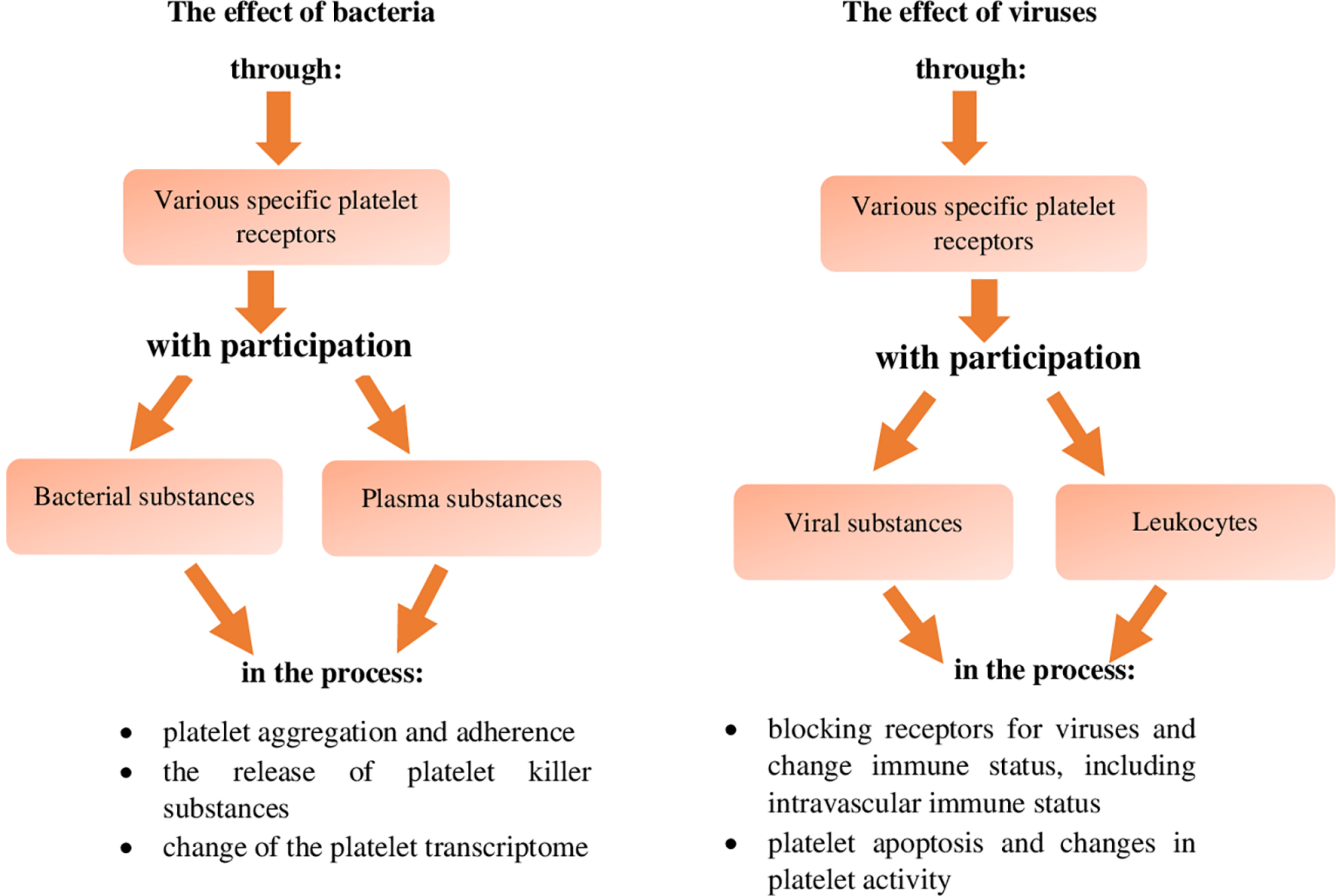
The effect of bacteria and viruses themselves on platelets leading to the inactivation of these pathogens (references in the text).

When describing the direct and indirect pathway of the destructive effect of platelets on bacteria, it is also important to explain the pathways of the effect of bacteria on platelets (**Figure 2**) in order to stimulate the activity of these cells to neutralise them (34, 62). This effect of bacteria on platelets can be direct and mediated by bacterial substances or it can occur indirect with the involvement of plasma proteins, which leads to aggregation and adhesion of platelets to the bacteria and consequently their immobilisation and neutralisation (31, 59). This pathway of bacterial effect on platelets involves the secretion of ClfA and B (clumping factors A and B) by bacteria during staphylococcal infection, which form bridges together with integrin receptors GPIIb and GPIIIa of platelets, fibrinogen, fibrin and thrombospondin thus immobilise bacteria (20, 58, 81, 87).

This effect of bacteria on platelets also includes the lysis of platelets caused by α-haemolysin of staphylococci, which leads to the release of biologically active substances from platelets, including those activating and increasing antibacterial immunity (20). It has been demonstrated that in the case of *Klebsiella pneumoniae* infection, the effect of these bacteria on platelets *via* the platelet TLRs and integrin receptor GP-VI leads to the secretion of TNF-α and chemokines CXCL-1 (MIP-2) and CCL-2 (MCP-1) by platelets, affecting the bactericidal activity of blood leukocytes, which enhances antimicrobial immunity (76, 82). In addition, phospholipase C secreted by *Pseudomonas aeruginosa* protease, as well as by *Proteus* sp., *Porphyromonas gingivalis* and *S. pyogenes*, affects platelets *via* the PAR-1 and PAR-4 receptor, and causes increased secretion of antibacterial substances (31, 87). *Listeria monocytogenes* infection also activates platelets to secrete substances increasing the activity of cellular elements of the immune system in the blood from their granules (11). Similarly, *Bordatella pertrusis*, affecting platelets *via* the ADP receptor, and *S. gordoni*, *S. sanguinis* and *Staph. aureus*, *via* the integrin marker GPIb, activate platelets to aggregate with these bacteria, leading to their neutralisation (31, 82). In the case of platelet aggregation related to *S. sanguinis* infection, this process is mediated not only by the integrin receptors GPIb (31, 82), but also by GPIIb/IIIa (81), and in the case of *Staph. aureus* by secreted coagulase (81). On the other hand, aggregation of enterococci and their immobilisation by platelets is mediated by "aggregating substances": pCF10 and PrpA (proline-rich protein A) (81). In addition to the activating effect of bacteria on the destructive activity of platelets, their inhibitory effect has also been described, because it has been demonstrated that during *Bacillus antrhracis* and *Staph. aureus* infection, adenylyl cyclase released from these bacteria, reduces the aggregation and secretory activity of platelets (17, 87). Fibrinogen-binding proteins, secreted during *Staph. aureus* and *S. pyogenes* infection and in the case of group C streptococci vWF, also reduce the antibacterial activity of platelets (43, 62). Moreover, in the case of *Staph. aureus* infection, it has been reported that α-haemolysin of this bacterium causes an inhibitory antibacterial effect of platelets, mainly due to a change in the activity of proteins of the MMP-ADAM-10 family (43). It has also been described that during bacterial sepsis, caused by streptococcal infection, as a result of molecular mimicry of collagen structural domains, the transcriptome of platelets changes, which may lead to a decrease in their activity, including antibacterial one (43, 62). In addition, during *E.coli* and *Staph. aureus* infections, the suppressive expression of genes of adhesion molecules of these cells *via* TLRs takes place, which reduces their proinflammatory and antibacterial activity (10). Moreover, during *Staph. aureus* infection, the degradation of BCL-xL (B-cell lymphoma-extra-large) proteins induces apoptosis of platelets, resulting in a state of thrombocytopaenia, which may lead to weakening of antibacterial immunity (10, 31, 62).

## Platelets and Viral Infections

**Figure 3:**
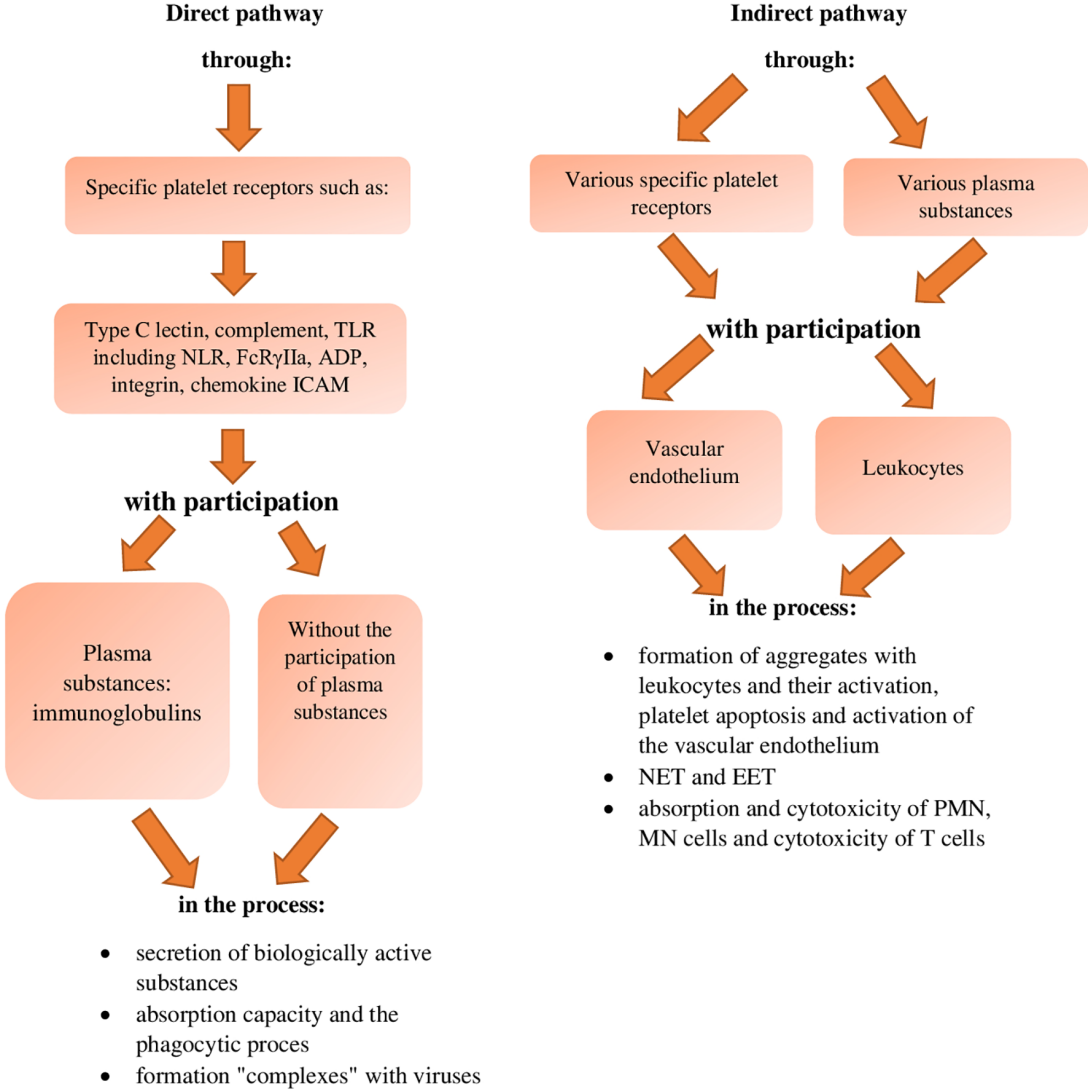
Inactivating effect of platelets against viruses (references in the text). TLR, Toll like-receptors; NLR, nucleotide-binding oligomerization domain-like receptors; ADP, Adenosine diphosphate receptor; ICAM, intracellular adhesion molecule.

The involvement and role of platelets in antiviral defence, similarly to antibacterial defence occurs *via* their direct activity, or interaction between specific platelet receptors and viruses without plasma substances and with the involvement of plasma substances (**Figure 3**) (43). On the other hand, the indirect pathway of the destructive effect of platelets on viruses occurs as a result of activation of blood leukocytes or vascular endothelium by these cells, *via* numerous different receptors of platelets and/or plasma substances (**Figure 3**) (43).

The direct neutralisation of viruses by platelets as compared to bacteria has been described in fewer sources and it is present to a lesser extent. However, as in the direct pathway of neutralising bacteria by platelets, the cooperation of these cells with viruses occurs *via* a wider range of their specific receptors, which are: - C-type lectin receptors, - complement receptors, - TLRs, including NLR, - FcRɣIIa, - ADP, - integrin receptors, - chemokine receptors, and - ICAM markers as well as *via* the same range of specific receptors, together with plasma substances which, unlike in the case of bacterial infections, are essentially only class-G serum immunoglobulins (42–44, 47, 76, 82, 96). These interactions result in the destruction of viruses by platelets due to (**Figure 3**):

- secretion of biologically active substances
- ability to absorb and phagocytosis process
- formation of "complexes" with viruses (references in the text).

The first pathway of direct activity of platelets is based on the secretion of virucidal compounds, e.g. defensins and chemokines by platelets, which have been reported in the case of encephalomyocarditis virus and HIV (Human immunodeficiency virus), and this reaction occurs *via* TLRs, but in the case of HIV mainly *via* the TLR-9 receptor (1, 11, 17, 75, 83, 96, 100, 114). It has also been reported (62) that during HIV infection, the platelet transcript may be altered to increase the synthesis of virucidal substances. During infection with this virus, the interaction of these factors with platelets *via* the C3a, C5a receptors induces the secretion of complement components by these cells, which increases the natural immunity, including antiviral immunity (1, 17, 44). During this infection, the chemokine CXC4 (PF-4) is also secreted from the EVs of platelets, which leads to inhibition of hepatic cell apoptosis and increase in the possibility of spreading HIV (51). During this infection, the penetration of HIV into hepatic cells is inhibited following the interaction of the aforementioned chemokine PF-4 (CXC4) as well as RANTES (CCL-5) secreted by platelets (82).

This pathway of neutralising viruses by platelets, in the case of infection with Dengue virus, is also associated with the virucidal substances of these cells, or type I IFN – resulting from the expression of IFITM-3 (interferon induced transmembrane -3) particles during the activation of platelets by this virus *via* the CLEC-2 receptor (C-type lectin-like receptor – 2), DC-SIGN (Dendritic Cell-Specific Intercellular adhesion molecule-3-Grabbing Non-integrin) (47, 133), as well as TLR-9 (83) and TLR-4 (82). Other studies (43, 76, 134) have shown that activation of platelets by the cytokine receptor during Dengue virus and HIV infection leads to the synthesis of IFN-α, IFN-β, IL-10 and IL-4 by these cells and activation of T regulatory cells as well as secretion of biologically active substances derived from the EVs of these cells (44, 113). On the other hand, during the human infection with HAV (hepatitis A virus), substances secreted from platelets have a protective effect on uninfected liver cells, which hinders the spread of this infection (1, 11, 51). It has been reported (1, 10, 11, 33, 34, 44, 62, 76, 82) that the Rift Valley fever virus, Hantan, Ebola as well as Crimean–Congo haemorrhagic fever and cowpox viruses activate platelets to secrete virucidal substances *via* TLRs, including NLRs and FcɣRIIa as well as CLEC-2 and DC-SIGN receptors. This pathway of neutralisation by platelets, by secreting virucidal substances, also applies to influenza virus, cytomegalovirus and HCV (Hepatitis C virus) (1, 17, 44, 47, 76, 82, 83, 96) and is mediated by the TLR-2, 3, 7, 9 receptors and the integrin marker as well as receptors for the complement components C3a and C5a, while in the case of Coxsackievirus by TLR-9 receptors (83). In addition, neutralisation by secreted virucidal substances, is mediated by the ADP receptor, while in the case of rotaviruses by the integrin marker GPIa/IIa-VLA2 (α2β1), and in the case of Hantan virus by the integrin receptor GPIIb/IIIa (αIIbβ3) (1, 10, 11, 33, 34, 62, 76). Other studies have demonstrated that when HCV and influenza are inactivated by platelets using this route, they interact *via* the TLR-9 marker.

On the other hand, the second pathway of the direct effect of platelets on viruses based on their absorption capacity and the process of phagocytosis is similar to analogous processes in bacterial infections, although it is less known (21, 33, 37, 43, 62, 67). The process of virus absorption by platelets was recorded in the case of Dengue virus, HCV, HSV-1 (herpes simplex virus A), smallpox (33, 42, 43, 62, 65), as well as cytomegalovirus, Coxsackievirus B and encephalomyocarditis virus (135), which are infections in case of which there no indications concerning receptors of platelet reaction were specified. On the other hand, the platelet phagocytosis process was observed in the case of HIV, influenza, SARS-CoV-2, leukaemia, measles as well as viruses of Newcastle disease, smallpox and herpes viruses (HSV-1) and it is mediated mainly by TLR-7 receptors (33, 43, 65, 67, 83, 100). In the case of HIV, the phagocytosed virus is found in the vesicles of endocytic vesicles of platelets along with the PMPs and kinocidins, and its unchanged form is observed in the case of influenza virus, the phagocytised virus loses in this process its integrity (33, 43). HIV infection has been shown to increase platelet phagocytosis by altering the expression of P-selectin and phosphatidylserine on platelets (33). It has been reported that, both in the process of absorption and in the process of phagocytosis of viruses by platelets, individual virus particles enter these cells, mainly *via* β-3 integrin receptors (33), while "clustered" viruses *via* type C lectin receptors, complement, chemokines, ICAM, FcɣRIIa and TLRs (117).

The third pathway of direct platelet effect on viruses is the pathway based on "complexes" formed with viruses by these cells, as described in the case of influenza and Dengue viruses, and it has been shown that this reaction is mediated by TLRs and FcɣRIIa, leading to thrombocytopaenia (1, 17, 62, 65, 76, 82), which is a condition affecting the immune status of the body (43, 67, 82, 83). This condition of thrombocytopaenia also arises as a result of the "complexes" formed with viruses by platelets, which was recorded in people infected with the MERS (middle east respiratory syndrome), SARS-CoV-2 (severe acute respiratory syndrome coronavirus 2) virus, RSV (respiratory syncytial virus), rotaviruses as well as some viruses of haemorrhagic fever (VHF - viral haemorrhagic fever), and in rabbits infected with GI.2-RHDV2/b virus (rabbit haemorrhagic disease virus) (1, 62, 65, 96, 136–139). It has also been reported that the platelet/influenza virus "complex" is formed using the platelet GPIIb/IIIa marker and induces thrombin signalling in these cells as well as increases the release of EVs from these cells, which enhances antiviral immunity (83). It has also been found that during infection with influenza virus, Dengue virus and HIV (1, 40, 48, 65), a large number of platelet/virus "complexes" increase the immune status, manifested by an increased pool of immune and inflammatory mediators, and during HIV infection, it additionally increases platelet volume (1, 40). It has also been reported that the condition of thrombocytopaenia, resulting from the formation of platelet/virus "complexes", also arises during the human infection with Varicella zoster virus as a result of antigenic mimicry of certain microorganisms and platelet GPIIIa integrin receptors (62). It is assumed that thrombocytopaenia as a result of the formation of platelet/virus complexes is the effect of increased activity of platelets, leading to their breakdown and the release of biologically active substances from their granules, including cytokines, growth factors, MMPs, histamine, serotonin, and catecholamines (139). These substances have, among others, a diastolic effect on blood vessels and an activating effect on the recruited cellular elements of blood immunity, thus enhancing the status of antiviral immunity (1, 2, 8, 10, 17, 36, 43, 46, 48, 58, 62, 74, 75, 96, 126). It should be added that this condition can also "trigger" the "coagulation system" not always favourable for endovascular homeostasis (43, 45). In addition to the recorded thrombocytopaenia as a result of the formation of platelet/virus complexes, it has been described that during infections with CCHF (Crimean-Congo haemorrhagic fever virus), Phleboviruses, Hantnaviruses, Coxsackievirus B in humans and with LCM (Lymphocytic Choriomeningitis) virus in mice, platelet/virus complexes cause thrombocytosis, a condition which prolongs the lives of infected individuals in the case of infections with CCHF viruses in humans and LCM virus in mice (1, 10), indicating that this is associated with an increased immune status, as in the case of a severe course of COVID-19, the increase in the number of platelets increases patient's chances of survival (140).

On the other hand, the indirect destructive effect of platelets on viruses is associated with their effect on blood leukocytes and the endothelium of blood vessels and occurs, as in the case of bacterial infections, *via* various receptors and *via* various biologically active substances in the plasma (43, 44), which leads to the neutralisation of viruses as a result of (**Figure 3**):

- the formation of aggregates with leukocytes and their activation, platelet apoptosis and activation of the vascular endothelium
- the activity of the NET network and EET network
- the process of absorption and cytotoxicity of PMN and MN cells and the process of cytotoxicity of T cells (references in the text). The pathway of indirect neutralisation of viruses by platelets, *via* the formation of platelet/neutrophil and platelet/monocyte aggregates, leads to an increased recruitment of blood immune cells and their activation for the secretion of virucidal substances, which was observed in the case of the infections with flavi-, filo-, bunya-, and arenaviruses, as well as influenza virus, HIV, cytomegalovirus, SARS-CoV-2 and viral sepsis (1, 10, 11, 39, 42, 44, 60, 65, 76, 78, 83, 85, 96, 97, 115, 141). In the case of adenovirus and HIV infections, aggregates of platelets with leukocytes, affecting B cells, increase the synthesis of serum IgG neutralising these viruses (1, 23, 62). Moreover, in this indirect way of neutralising viruses by platelets, it has been shown (40) that the platelet/monocyte aggregates formed during Dengue virus infection, synthesising the MIF (migration inhibitor factor) lymphokine, stimulate monocytes to greater biogenesis of lipid droplets – functional and active elements of the body in physiological and pathological state, and secretion of inflammatory mediators by cells of the immune system (40). Also during the indirect effect of platelets on the SARS-CoV-2 and Denga viruses, the resulting platelet/monocyte aggregates activate monocytes in the area of motion and secretion of TF, which increases their virucidal effect against these viruses (10, 39, 44, 101). Also in the case of this pathway of the effect of platelets on viruses, it has been reported that during Dengue virus infection, the resulting aggregates of platelets with monocytes activate PMN cells and macrophages for the secretion of proinflammatory cytokines (39, 47). An analogous picture of the increase in immunity caused by the formation of aggregates with blood leukocytes by platelets was observed during infection with influenza virus and unspecified viruses (1, 11, 83, 94, 96, 135). It has been shown that the recorded increase in antiviral immunity is conditioned by the expression of platelet CD40 receptor, activation of P-selectin, PAF factor and increased, thanks to the PSGL-1 ligand (P-selectin glycoprotein ligand-1), neutrophil adhesion capacity, as well as increased aggregation and secretion of leukocytes in terms of chemokines and cytokines (1, 11, 83, 94, 96, 135). In contrast, in the case of adenovirus infections, aggregation of platelets with leukocytes, leading to a change in the expression of the CD40 and CD40L marker on platelets, changes the synthesis of IgG isotypes in B cells, thereby enhancing the neutralisation of these viruses (62, 82). It is assumed that the CD40L receptors of activated platelets also have an immunomodulatory effect, mainly on natural immunity, including antiviral immunity (82). Therefore, during various viral infections (39, 43), as a result of the formation of platelet/leukocyte aggregates, serotonin secreted from platelets induces immune cells to increase the secretion of antiviral substances. It has also been shown that the mutual communication between cells forming platelet/leukocyte aggregates during this pathway of Coxsackievirus B, HAV and HCV neutralisation by platelets, and the emerging NLRP-3 inflammasome, enhance the secretion of IL-1β and IL-18, which affects immunity status, including natural immunity (37, 51, 82). Such a condition was also recorded in the case of Dengue virus infection, in which the interaction of platelets with leukocytes occurs *via* the TLR-4 receptor (47, 82). This indirect pathway of the effect of platelets on viruses is also associated with their apoptosis, during which the resulting reduced mitochondrial potential of platelets and increased expression of their surface phosphatidylserine increases the virucidal properties of cellular immune elements in the blood, which was recorded in Dengue virus infection (35). Another important process in the pathway of the indirect effect of platelets on viruses is the activation of the vascular endothelium. It has been reported that during SARS-CoV-2 infection, chemokines released from platelets increase the permeability of blood vessels and increase the inflow of immune cells (47, 101), including T regulatory cells and B cells in which the switching of IgA isotype- mainly SIgA takes place (10, 17, 39, 62, 65). This condition also increases the expression of MHC class I molecules and the receptor for complement C-3 and C-7 on cells of the immune system (10, 17, 39, 62, 65).

Furthermore, the pathway of indirect virus neutralisation by platelets, mediated by the NET network, is one of the most effective processes of neutralising these pathogens, which is less known in terms of molecular mechanisms, compared to the NET network present in bacterial infections. It has been shown that this reaction, together with vascular endothelial cells, can also activate factors in the coagulation pathway to form fibrin, leading to a process known as "immune thrombocytopaenia" (1, 2, 10, 11, 17, 42–45, 65, 101, 111, 112), which is a condition reducing organ damage by limiting pathogen neutralisation (101). This indirect pathway of the effect of platelets on viruses, mediated by the NET network, has been observed in the case of numerous viruses and the virus/platelet reaction occurs *via* various receptors. It has been shown that during human infection with cytomegalovirus, this reaction occurs *via* the TLR-2 (1), in the case of SARS-CoV-2 *via* the TLR-7 marker (65), in the case of poxviruses *via* TLR-2,3,4,6 and 7 (1, 95, 96, 116), in the case of influenza virus *via* the TLR-7 and FcRɣIIa marker (1, 10, 96), and in rabbits infected with myxomatosis virus *via* the TLR-3 receptor (2). In unspecified viral infections, TLR-1 (1) receptors are platelet receptors in this pathway of the antiviral activity, while it is assumed that in the case of ssRNA single-stranded RNA viruses, this role is played by TLR-7 markers (96). During the infection of humans with bunyaviruses and HCV, the interaction of platelets in this indirect pathway *via* the NET network is mediated by FcRɣIIa receptors, and in the case of Dengue virus by glycosylation of these receptors, which leads to a change in the quantitative ratio between IgG1 and IgG2 and the CLEC-2, CLEC-3, DC-SIGN, TLR-4 and TLR-2 markers (1, 10, 37, 39). In the case of the indirect pathway of the effect of platelets on viruses *via* the EET network, the reaction is mediated by the CLEC-2 and TLR-7 receptors, which activates the platelets for release of substances from their EVs, which stimulates eosinophils to secrete virucidal compounds, as it was recorded in influenza virus infection (45).

The third indirect way of the destructive platelet effect on viruses is their influence on the ability to absorb PMN and MN cells, which was described for the infection with influenza virus (83). In this process of absorption of influenza viruses by PMN and MN cells, they are not only neutralised, but additionally this reaction activates platelets, *via* their TLR-7 markers, to increased expression of the complement C-3 receptor, which additionally enhances antiviral immunity. conditioned by proteins of the complement system (83). The process of absorption and cytotoxicity of PMN and MN cells as well as cytotoxicity of T cells, as a result of their activation by platelets, were observed during infection with HBV (hepatitis B virus) (1, 11, 17, 26). The process of macrophage cytotoxicity during human infection with HAV and HCV, EBV (Epstein-Barr virus), herpesvirus, HIV and Coxsackievirus B has also been described as a part of indirect pathway of the destructive effect of platelets on viruses (10, 11, 35, 51).

When analysing the direct and indirect destructive effect of platelets on viruses, it should be stated that, similarly to bacteria, viruses also affect platelets (**Figure 2**), as reported in the case of HIV (33) and SARS-CoV-2 (142) in humans, as well as in rabbits in the case of myxoma virus, and in poultry in the case of Newcastle disease virus (43). It has been shown that the Tat protein secreted by HIV, interacting *via* the chemokine receptors - CCL-3 (MIP-12) and β-3 integrin of platelets, activates them to block HIV receptors on leukocytes, thus reducing its spread (33). In turn, neuraminidase of this virus, affects platelets (33), and therefore promotes their hepatic clearance, which indirectly reduces the state of antiviral immunity and promotes its infectivity. During HIV infection, the activation of caspases and apoptosis of platelets is also mediated by cytochrome C, which leads to a reduction in their number, and thus weakening of antiviral immunity (77). Similarly, the SARS-CoV-2 virus (142), by directly affecting PMN cells, promotes the damage and death of vascular endothelial cells, which lowers the state of intravascular immunity and increases the potential for the spread of this virus. A similar inhibitory effect on antiviral immunity was recorded in the infection of rabbits with myxoma virus and in poultry with Newcastle disease virus (43), because it has been shown that neuraminidase of the rabbit myxomatosis virus, cleaves the membrane of platelets, and thus reduces their viability and activity, and Newcastle disease virus damages the platelet membrane, and causes changes in their number, which in turn leads to the weakening of antiviral immunity in these infections.

## Summary

As a result of their proinflammatory effects, including antibacterial and antiviral properties, platelets are important elements determining resistance and intravascular immunity. Moreover, as a result of interaction with the cells of the blood immune system, removing bacteria and viruses in various ways, both directly and indirectly, they condition a specific intravascular homeostasis in the field of innate and acquired anti-infective immunity.

## Author Contributions

All authors contributed to the article and approved the submitted version.

## Conflict of Interest

The authors declare that the research was conducted in the absence of any commercial or financial relationships that could be construed as a potential conflict of interest.

## Publisher’s Note

All claims expressed in this article are solely those of the authors and do not necessarily represent those of their affiliated organizations, or those of the publisher, the editors and the reviewers. Any product that may be evaluated in this article, or claim that may be made by its manufacturer, is not guaranteed or endorsed by the publisher.
